# Supplementary material for: River temperature response to atmospheric heatwaves is modulated by discharge and meltwater
Source: Commun Earth Environ. 2026 Feb 19;7(1):296. doi: 10.1038/s43247-026-03269-6 (PMC13038413; doi:10.1038/s43247-026-03269-6)
Supplement: Supplementary file 2 — Supplemantary material [file 43247_2026_3269_MOESM2_ESM.pdf]

Supplementary information for

River temperature response to atmospheric heatwaves is modulated by discharge and meltwater

Amber van Hamel[1,2,3], Joren Janzing[1,2,3], and Manuela Irene Brunner[2,1,3]

1 Supplementary Methods: Details on the identified atmospheric and riverine heatwaves

In total, we identified 5145 atmospheric heatwaves during the period 2011-2021 (average of 19 per station). Of those, 74% had a duration of 5 to 6 days and only 1.8% (n =93) of the heatwaves had a duration of more than 10 days. The longest atmospheric heatwave lasted for 14 days and occurred in November 2015 at a small and low elevation catchment on the Southern side of the Alps (station CH_2612).

The number of identified riverine heatwaves (n =7199) is larger than the number of atmospheric heatwaves (n =5145), with an average of 26 (min 14, max 39) riverine heatwaves per station. Since the same definition is used to identify both riverine and atmospheric heatwaves, this suggests that daily water temperature variability is lower than air temperature variability, meaning that water temperature more easily meets the minimum duration criterion of five days. This is also supported by the average duration of the riverine heatwaves, which is 8 days on average, and thus longer than the average duration of atmospheric heatwaves (5–6 days). Around 15% of the riverine heatwaves lasted more than 10 days, and 5% lasted more than 2 weeks (14 days). The longest riverine heatwave lasted 89 days (3 months) and occurred in the winter of 2014-15 at station AU_212373. This station is located along the Möll river, close to Winklern and the river, which originates from a glacier in the National Park Hohe Tauern, and is strongly impacted by water withdrawals. In total, 2266 riverine heatwaves overlapped, at least in part, with an atmospheric heatwave, accounting for around 31% of the riverine heatwaves. The remaining riverine heatwaves could not be directly linked to atmospheric heatwaves.

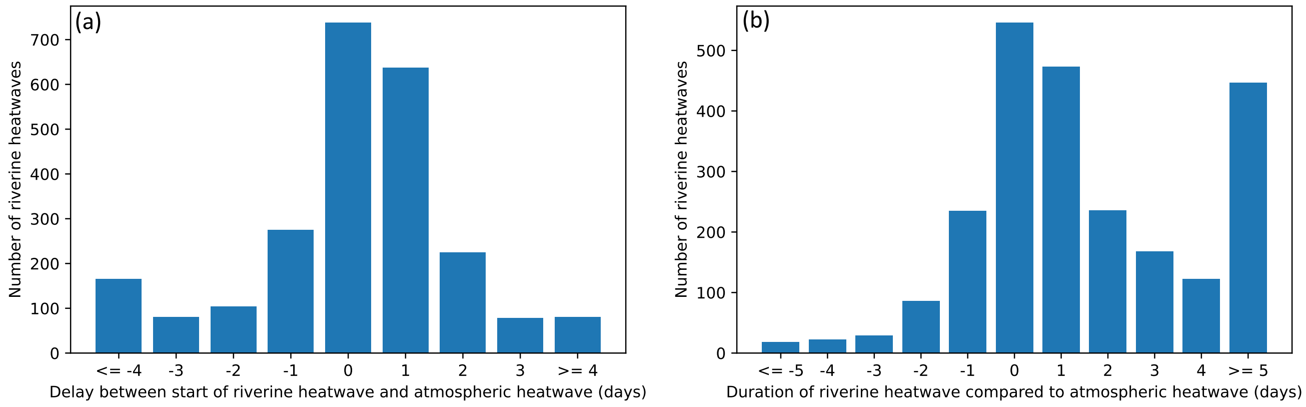

Supplementary Figure 1: **Riverine heatwave start day and duration compared to the atmospheric heatwave with which they co-occur.** **(a)** Starting day of the riverine heatwave, before (negative), same day, or after (positive), compared to the starting day of the overlapping atmospheric heatwave. **(b)** Duration in days of the riverine heatwave, longer (positive), same duration, or shorter (negative), compared to the duration of the overlapping atmospheric heatwave.

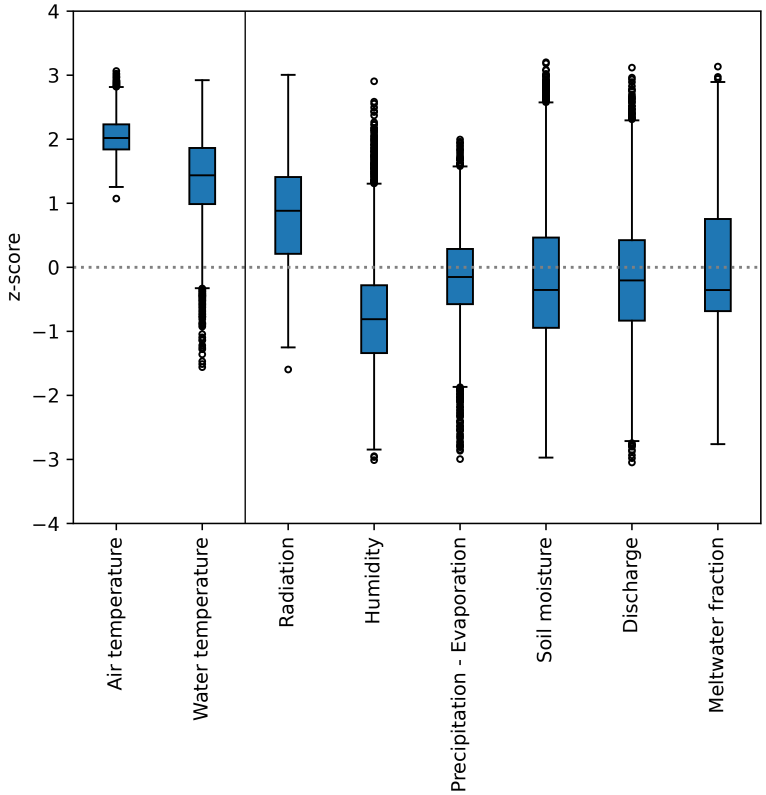

Supplementary Figure 2: **Z-scores of different hydro-climatic variables during atmospheric heatwaves.** The boxes represent the interquartile range, with the line indicating the median. The whiskers extend to points up to 1.5 times the box range. Outliers are plotted as individual points beyond the whiskers.

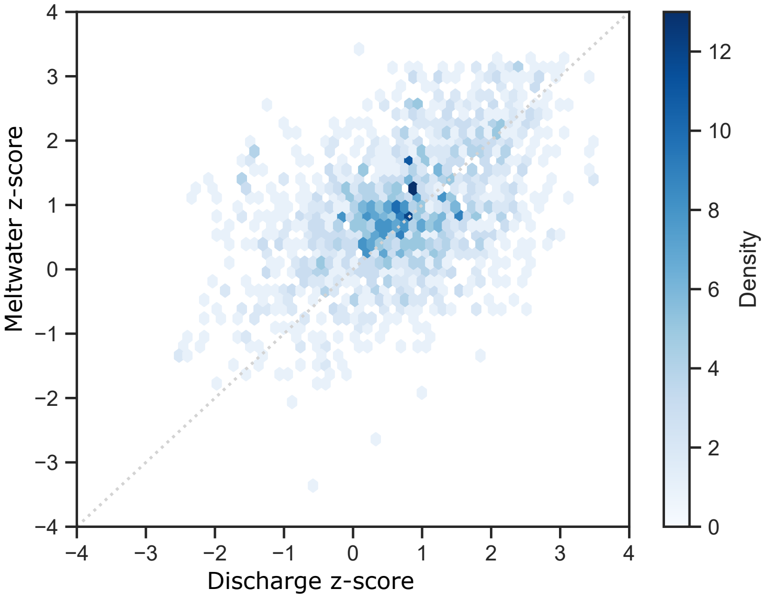

Supplementary Figure 3: **Density plot of discharge and meltwater z-scores.** Discharge z-scores (x-axis) against meltwater z-scores (y-axis) presented by hexagonal bins to represent the density of data points within the two-dimensional space.

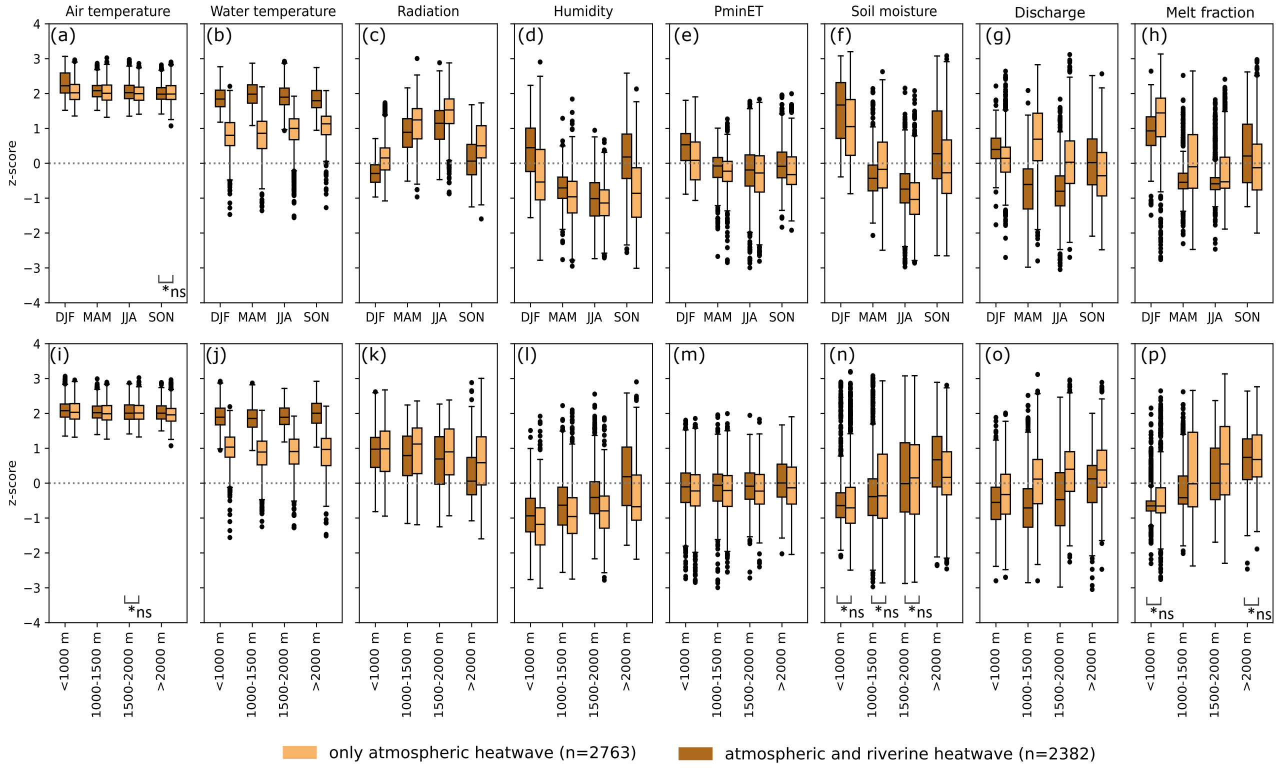

Supplementary Figure 4: **Z-scores of different hydro-climatic variables during atmospheric heatwaves grouped by season and elevation.** The hydro-climatic variables per season **(a)-(h)** and by elevation band **(i)-(p)**. The light brown boxplots show cases where atmospheric heatwaves do not result in riverine heatwaves, characterized by much lower water temperature z-scores. The dark brown boxplots show cases where atmospheric heatwaves result in riverine heatwaves, characterized by high water temperature z-scores. The boxes represent the interquartile range, with the line indicating the median. The whiskers extend to points up to 1.5 times the box range. Outliers are plotted as individual points beyond the whiskers. Pairs that are marked with *ns did not show significant statistical difference based on the Mann-Whitney U Test ($p>0.05$).

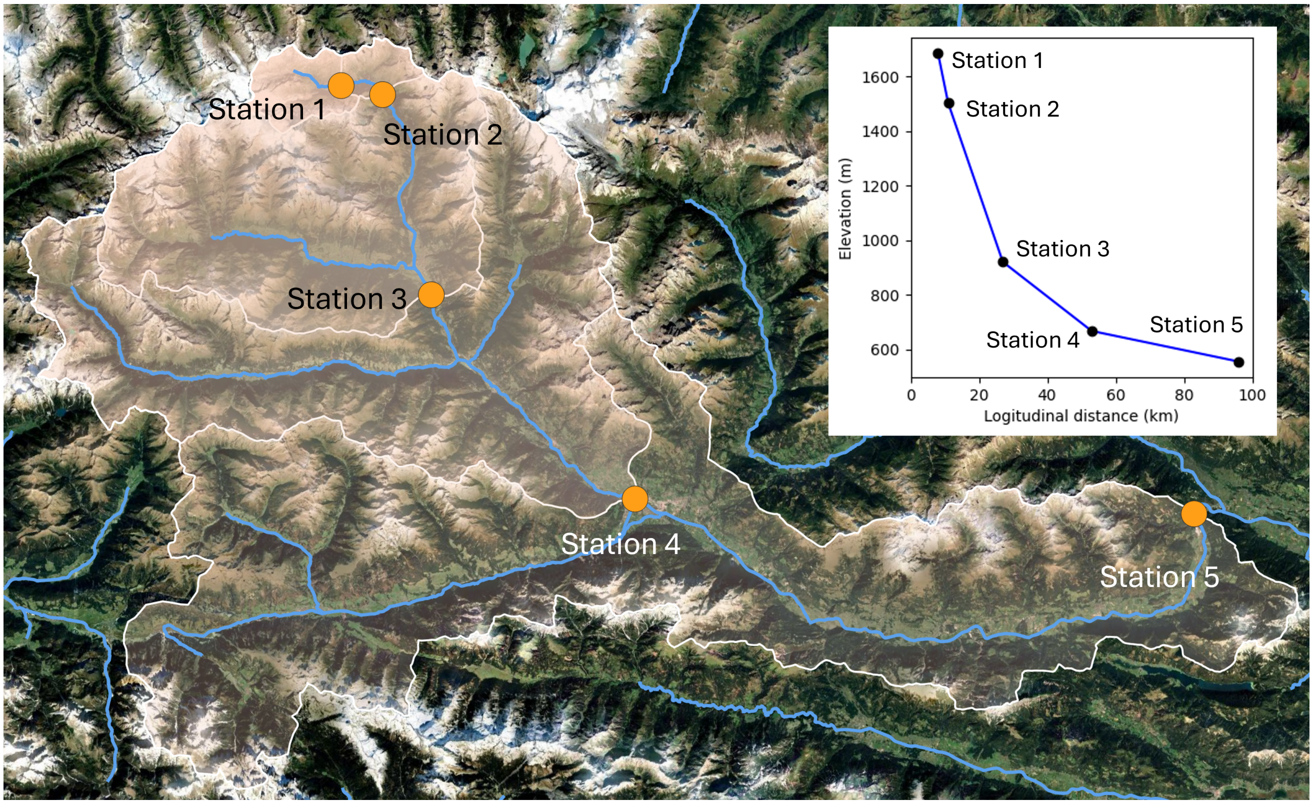

Supplementary Figure 5: **Five stations along the Drau River.** This river has its source at the glaciers of the Großvenediger mountain in the National Park 'Hohe Tauern' (South-eastern Austria). The highest measurement station (Station 1) is located at 1686 m.a.s.l. and the lowest (Station 5) at 555 m.a.s.l. As a result, the catchment covers an elevation range of 1131 m over a vertical distance of approximately 100 km. Images/Maps data: © OpenStreetMap Foundation (CC-BY-SA).

Supplementary Table 1: **Additional information on the five selected lakes.** Overview of the lake surface area, type of regulation, and the distance of the upstream and downstream stations to the lakes.

**Lake Biel**	
Lake surface area	39 km^2
Natural/ regulated	Regulation of the water levels of Lake Biel is controlled in coordination with the lake Neuchâtel and lake Murten. The regulation allows for an annual cycle with seasonal variations in water level. Average discharge at the lake outlet: 241 m^3/s. Highest measured discharge: 761 m^3/s.
Distance from upstream station	0.8 km
Distance to downstream station	4.5 km
Hydrological regime	Nival regime (at both the up- and downstream station)
**Lake Brienz**	
Lake surface area	29.8 km^2
Natural/ regulated	Regulation of the lake outflow 2 km downstream of lake outlet. The outflow of lake Brienz flows through two small hydropower plants (Mühle Burgholz and Livta), and when discharge exceeds 26 m^3/s also through the gates of one big weir. Another smaller weir can be opened during exceptionally high outflows. Lake regulation remains limited to periods of exceptionally high water levels. Average discharge at the lake outlet: 62 m^3/s. Highest measured discharge: 344 m^3/s.
Distance from upstream station	3.4 km
Distance to downstream station	0 km, directly at lake outlet and upstream of regulation structures
Hydrological regime	Nival regime (both for the up- and downstream station)
**Lake Geneva**	
Lake surface area	581 km^2
Natural/ regulated	Lake Geneva's water level is regulated by the Seujet Dam in Geneva, which controls the outflow to the Rhône river. The dam maintains the average surface elevation at approximately 372 m.a.s.l. while it also manages the outflow to balance competing demands such as flood control and water supply. The overall management of the lake is a collaborative effort between Switzerland and France through the International Commission for the Protection of the Waters of Lake Geneva (CIPEL).
Distance from upstream station	6 km
Distance to downstream station	0 km, directly at the lake outlet
Hydrological regime	Nival regime (both for the up- and downstream station)
**Lake Lucerne**	
Lake surface area	114 km^2
Natural/ regulated	According to the weir regulations, water level fluctuations in Lake Lucerne are permitted as in an unregulated lake. The regulation ensures a largely natural water level regime within certain tolerance limits. Regulation is only applied to prevent the lake level from falling below 433.45 m.a.s.l. or reaching above 434 m.a.s.l. Average discharge at the lake outlet: 110 m^3/s. Highest measured discharge: 473 m^3/s.
Distance from upstream station	1.8 km
Distance to downstream station	1 km (just downstream of the weirs)
Hydrological regime	Nival regime (both for the up- and downstream station)
**Lake Walen**	
Lake surface area	24 km^2
Natural/ regulated	Natural (no lake level regulation)
Distance from upstream station	5 km
Distance to downstream station	0.8 km
Hydrological regime	Nival regime (both for the up- and downstream station)
